# Safety and Efficacy of Rapid Primary Phacoemulsification on Acute Primary Angle Closure with and without Preoperative IOP-Lowering Medication

**DOI:** 10.3390/jcm12020660

**Published:** 2023-01-13

**Authors:** Takafumi Suzuki, Takashi Fujishiro, Naoko Tachi, Yoshiki Ueta, Takao Fukutome, Yasuhiro Okamoto, Hirofumi Sasajima, Makoto Aihara

**Affiliations:** 1 Department of Ophthalmology, Shinseikai Toyama Hospital, Toyama 939-0243, Japan; 2 Department of Ophthalmology, The University of Tokyo Hospital, Tokyo 113-8655, Japan

**Keywords:** acute primary angle closure, phacoemulsification, best-corrected visual acuity, intraocular pressure

## Abstract

This study aimed to investigate the safety and efficacy of rapid primary phacoemulsification in patients with acute primary angle closure (APAC) (*n* = 41), with or without preoperative IOP-lowering medication. The best-corrected visual acuity (BCVA), intraocular pressure (IOP), number of corneal endothelial cells (CECs), and number of IOP-lowering medications at the preoperative examination (Pre), postoperative day 1 (1d), week 1 (1w), and month 1 (1m) were used as indicators and compared. BCVA significantly improved at 1d, 1w, and 1m compared with Pre (*p* < 0.05) and significantly improved at 1m compared to 1d (*p* < 0.05) for all eyes. IOP significantly decreased at 1d, 1w, and 1m compared with Pre (*p* < 0.05). CECs were not significantly different between Pre and 1m; the number of IOP-lowering medications decreased significantly from Pre to 1m (*p* < 0.05). BCVA and IOP were not significantly different between the two groups for all periods. CECs were not significantly different between the two groups at Pre and 1m. Rapid primary phacoemulsification improved visual acuity due to improvement of corneal edema without central visual field defects and good IOP control without reoperation or IOP-lowering medication and maintained the number of corneal endothelial cells with or without preoperative IOP-lowering medication in patients with APAC.

## 1. Introduction

Glaucoma is a chronic disease that is initially asymptomatic but can cause progressive optic nerve damage. It is estimated that 76 million people worldwide suffer from glaucoma [1]. Glaucoma is a leading cause of irreversible blindness worldwide [2]. Although this damage is irreversible, further progression can be prevented or delayed by lowering the intraocular pressure (IOP).

Glaucoma leads to a reduction in the quality of life, which is directly related to health and economic losses. The prevalence of glaucoma among the elderly is 3.5% worldwide, and the number of glaucoma patients worldwide is expected to reach 111.8 million by 2040 [1].

Acute primary angle closure (APAC) is a disease requiring urgent treatment. Common treatments include medical therapy, such as miosis eye drops to remove the pupillary block and IOP-lowering eye drops or intravenous infusion, followed by laser peripheral iridotomy (LPI) or phacoemulsification to remove the pupillary block [3,4,5,6].

To the best of our knowledge, no reports have examined the course of very early phacoemulsification as a primary treatment for APAC at multiple points in detail and included a relatively large number of cases without preoperative IOP-lowering medication (glaucoma eye drops, oral medications, or intravenous infusion).

Therefore, we retrospectively examined the short-term results of very early phacoemulsification as the primary treatment for APAC at multiple points. We also retrospectively examined subgroups of patients with and without preoperative IOP-lowering medications.

## 2. Materials and Methods

### 2.1. Ethics

This study adhered to the tenets of the Declaration of Helsinki and was approved by the Institutional Review Board of Shinseikai Toyama Hospital (approval number: 220929-1). Our study was a retrospective consecutive series. We used an opt-out consent process, and the requirement for informed consent was waived by the institutional review board of Shinseikai Toyama Hospital.

### 2.2. Patients

The patient database at the Shinseikai Toyama Hospital was searched, and the records of patients with APAC who visited our hospital between June 2010 and June 2021 were reviewed. In total, 41 eyes from 41 patients were included in this study. The follow-up period was 1 month. The inclusion criteria were as follows:

1. Presence of a history of intermittent blurring of vision with halos and at least two of the following symptoms: ocular or periocular pain, nausea and/or vomiting.
2. IOP > 30 mmHg and the presence of at least three of the following signs: conjunctival injection, corneal epithelial edema, mid-dilated pupil, shallow anterior chamber, and iris atrophy.
3. Completion of more than 1 month of follow-up.

The exclusion criteria were as follows:

1. Secondary glaucoma, such as neovascular glaucoma, phacolytic glaucoma, and uveitic glaucoma.
2. Pseudophakic eyes, aphakic eyes, or eyes that underwent LPI.

### 2.3. Ophthalmic Examinations

All patients in this study underwent comprehensive examinations including best-corrected visual acuity (BCVA), IOP, specular microscopy, and slit-lamp biomicroscopy. They were followed up on day 1, at week 1, and one month after phacoemulsification.

All IOPs were measured using noncontact tonometry, and the average of three measurements was used.

### 2.4. Treatment Protocol

All patients underwent phacoemulsification within days after the onset of APAC. None of the patients underwent LPI.

After the arrival of the patient at the hospital, we completed the ophthalmic examination and tests necessary for surgery, initiated topical administration of tropicamide and phenylephrine instead of LPI, and performed phacoemulsification at the earliest. We administered IOP-lowering eye drops, acetazolamide, or glycerol systemically till the surgery if the patient experienced severe headache or nausea.

Phacoemulsification with IOL implantation was performed by two surgeons (NT and YU) under topical and sub-Tenon anesthesia. A 2.2 mm and superior scleral or corneal incisions was made. After phacoemulsification, a foldable 6.0 mm IOL (SN60WF; Alcon, Fort Worth, TX, USA or ZCB00V; AMO, Riverside, CA, USA) was implanted into the capsular bag. In addition, the anterior segment was refilled with viscoelastic and the angle was observed with a Mori goniotomy lens (R E MEDICAL Inc., Osaka, Japan) after the insertion of IOL. Goniosynechialysis (GSL) was performed without causing angle dissection if peripheral anterior synechiae (PAS) were visible. The surgeons placed a viscoelastic material near the PAS and gently pressed it against the peripheral iris with a blunt needle to exert backward pressure on the iris and expose the trabecular meshwork. The same procedure was performed using a Nagata GSL needle (Inami, Japan) if the PAS persisted. GSL was stopped before goniodialysis occurred if the PAS persisted even after performing these techniques.

Postoperative treatment included administering a combination of moxifloxacin 0.5% three times daily, fluorometholone 0.1% or betamethasone 0.1% four times daily, and bromfenac 0.1% twice daily, which was tapered over a month.

### 2.5. Evaluation of the Effectiveness of Rapid Phacoemulsification

After the preoperative examination (Pre) and surgery, the patients were followed-up on day 1 (1d), at week 1 (1w), and after month 1 (1m).

We examined the difference between the number of men and women; the time from APAC onset to the start of surgery; the time from arrival at the hospital to the start of surgery; BCVA and IOP at Pre, 1d, 1w, and 1m and the changes in the values; the presence of corneal epithelial edema at the start of surgery, surgery time, and perioperative complications; the number of IOP-lowering medications and corneal endothelial cells (CECs) at Pre and 1m and the change in the values from Pre to 1m; the correlation between IOP at Pre (Pre-IOP) and BCVA at Pre (Pre-BCVA); the correlation between Pre-IOP and BCVA at 1m (1m-BCVA); and the correlation between Pre-IOP and BCVA change from Pre to 1m (change in BCVA).

As a subgroup analysis for the procedure with and without preoperative IOP-lowering medications, we also compared the following parameters between the two groups: the difference in the ratio of men and women; the time from onset of APAC to the start of surgery; the time from arrival at the hospital to the start of surgery; BCVA and IOP at Pre, 1d, 1w, and 1m; the presence of corneal epithelial edema at the start of surgery; surgery time; perioperative complications; the number of IOP-lowering medications; and CECs at Pre and 1m.

### 2.6. Statistical Analysis

Continuous variables are presented as mean (±standard deviation).

An exact binomial test was performed to determine the differences in the number of males and females. BCVA was converted to the logarithm of the minimal angle of resolution. Tukey’s multiple comparison test was performed for BCVA and IOP at Pre, 1d, 1w, and 1m. The Wilcoxon signed-rank test was performed for the number of IOP-lowering medications and CECs at Pre and 1m.

Pearson’s correlation analysis was used to determine the correlation between pre-IOP and pre-BCVA, the correlation between pre-IOP and 1m-BCVA, and the correlation between pre-IOP and change in BCVA. Statistical significance was set at *p* < 0.05.

For subgroup analysis, Fisher’s exact test was used to determine whether a difference was present in the male-to-female ratio between the two groups. The Wilcoxon rank-sum test was used to compare age, time from onset of APAC to surgery, time from arrival at the hospital to surgery, and surgery time. The Wilcoxon rank-sum test was also used to compare BCVA and IOP at pre, 1d, 1w, and 1m. The Wilcoxon rank sum test was used to compare the number of IOP-lowering medications and CECs at Pre and 1m.

Statistical analyses were performed using R version 4.0.2 (The R Foundation for Statistical Computing, Vienna, Austria).

## 3. Results

### 3.1. Patient Characteristics

In total, 41 eyes from 41 patients were included in this study. The characteristics of the patients are presented in Table 1. The patients included 9 men (9 pairs of eyes) and 32 women (32 pairs of eyes) (mean age, 73.7 ± 8.7 years). Two cases of pseudoexfoliation syndrome without lens dislocation and three cases of mild epiretinal membrane cellophane maculopathy. These comorbidities were not considered to have significantly confounded the study results. All patients underwent phacoemulsification within a day of their initial visit to our hospital. The mean time from the initial visit to our hospital to phacoemulsification was 5.9 ± 7.3 h. The mean time from onset to phacoemulsification was 48.0 ± 46.6 h. An accurate and quantitative assessment of the degree and extent of PAS using gonioscopy during the ophthalmic examination was not possible due to corneal edema and symptoms such as headache or nausea.

The characteristics of a subgroup analysis are shown in Table 2.

In total, 26 pairs of eyes from 26 patients were included in the preoperative IOP-lowering medication group, including 6 men (6 pairs of eyes) and 20 women (20 pairs of eyes) (mean age, 73.0 ± 8.5 years). The mean time from onset to phacoemulsification was 48.5 ± 41.9 h, and the mean time from the initial visit to our hospital to phacoemulsification was 7.3 ± 8.5 h.

In total, 15 pairs of eyes of 15 patients were included in the group without preoperative IOP-lowering treatment, including 3 men (3 pairs of eyes) and 12 women (12 pairs of eyes) (mean age, 74.9 ± 8.9 years). The mean time from onset to phacoemulsification was 47.3 ± 53.8 h and the mean time from the initial visit to our hospital to phacoemulsification was 3.3 ± 3.5 h.

There were no significant differences in sex ratio (*p* = 1.0), age (*p* = 0.69), mean time from onset to phacoemulsification (*p* = 0.19), and mean time from the initial visit to our hospital to phacoemulsification (*p* = 0.06) between the two groups.

### 3.2. Operation and Complications

Among the 41 eye pairs, 30 eye pairs had corneal edema at the start of surgery. The operative time was 20.2 ± 7.9 min (Table 1).

The IOL was inserted into the bag in all cases; no severe adverse events were observed. Small surgical complications included iris prolapse in three eyes, anterior chamber hemorrhage in three eyes, consecutive rupture of the Zinn’s zonule within 45 degrees in one eye, continuous curvilinear capsulorhexis (CCC) tear (one location) in one eye, and iridodialysis to an extent of approximately 45 degrees in one eye. On postoperative day 1, 11 eyes had anterior chamber fibrin, and 4 eyes had anterior chamber hemorrhage, which improved over time. None of the patients required reoperation within one month (Table 3). An accurate and quantitative assessment of the degree and extent of PAS using a Mori goniotomy lens during surgery was not possible because of corneal edema. However, there were 28 eyes in which even a small extent of PAS was identified within the range visible with the lens.

Among the 26 eyes in the group with preoperative IOP-lowering medications, 20 eyes had corneal edema at the start of surgery. The operation time was 20.0 ± 7.9 min (Table 2). Small surgical complications included iris prolapse in 3 eyes, anterior chamber hemorrhage in 3 eyes, CCC tear (one location) in 1 eye, and iridodialysis to an extent of approximately 45 degrees in 1 eye. On postoperative day 2, 2 eyes had anterior chamber fibrin, and 1 eye had anterior chamber hemorrhage, which improved over time (Table 3).

Among the 15 eyes in the group that did not receive preoperative IOP-lowering medications, corneal edema was observed in 10 eyes at the start of the surgery. The operation time was 20.3 ± 7.9 min (Table 2). Small surgical complications included consecutive rupture of the Zinn’s zonule within 45 degrees in one eye. On postoperative day 1, 9 eyes had anterior chamber fibrin, and 3 eyes had anterior chamber hemorrhage, which improved over time (Table 3).

There was no significant difference in surgery time (*p* = 0.87) (Table 2).

### 3.3. Changes in BCVA, IOP, and the Number of CECs and IOP-Lowering Medications

**Figure 1:**
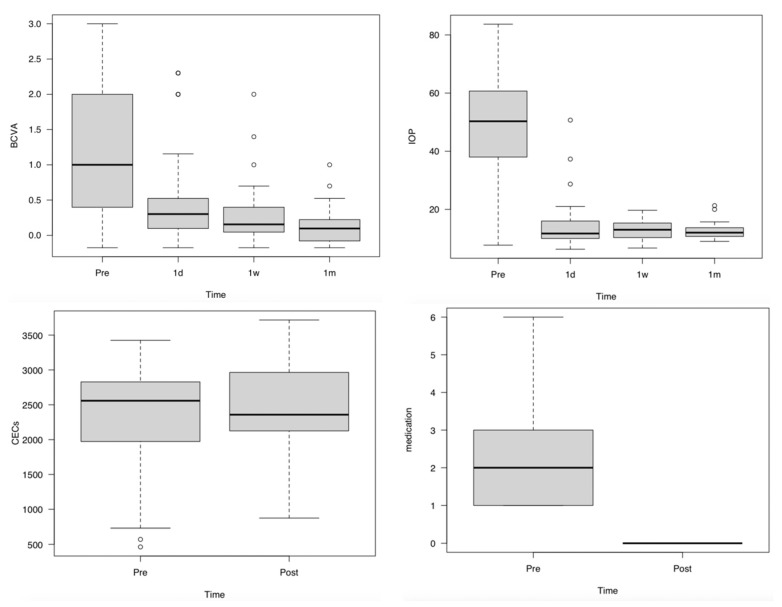
Change in BCVA, IOP, and the number of corneal endothelial cells. BCVA improved significantly for all 41 eyes at 1d, 1w, and 1m compared with that at Pre (*p* = 0.0002, *p* < 0.0001, and *p* < 0.0001, respectively) and significantly at 1m compared with that at 1d (*p* = 0.0061). IOP decreased significantly at 1d, 1w, and 1m compared with that at Pre (*p* < 0.0001, <0.0001, and <0.0001, respectively). CECs did differ not significantly between Pre and 1m (*p* = 0.784), and the number of IOP-lowering medications decreased significantly from Pre to 1m (*p* < 0.0001). Abbreviations: BCVA, LogMAR-converted best corrected visual acuity; IOP, intraocular pressure (mmHg); CECs, number of corneal epithelial cells (/mm^2^); medication, number of glaucoma medication; Pre, Preoperative period; 1d, The day after surgery; 1w, 1 week after surgery; and 1m Post, 1 month after surgery.

For all 41 pairs of eyes, BCVA significantly improved at 1d, 1w, and 1m compared with that at Pre (*p* = 0.0002, *p* < 0.0001, and *p* < 0.0001, respectively) and significantly improved at 1m compared with that at 1d (*p* = 0.0061). IOP significantly decreased at 1d, 1w, and 1m compared with that at Pre (*p* < 0.0001, <0.0001, and <0.0001, respectively). CECs did not differ significantly between Pre and 1m (*p* = 0.301), and the number of IOP-lowering medications decreased significantly from Pre to 1m (*p* < 0.0001) (Table 1) (Figure 1).

The results of the comparison of subgroups with and without preoperative IOP-lowering medications were as follows: BCVA was not significantly different between the two groups at 1d, 1w, and 1m (*p* = 0.12, 0.94, 0.30, and 0.15, respectively). IOP was not significantly different between the two groups at Pre, 1d, 1w, and 1m (*p* = 1, 0.27, 0.60, and 0.45, respectively). CECs did not differ significantly different between the two groups at Pre and 1m (*p* = 0.41, 0.69, respectively). The number of IOP-lowering medications was significantly different between the two groups at Pre (*p* < 0.0001), but not at 1m (*p* = not significant) (Table 2).

### 3.4. Correlation between Pre-IOP and Pre-BCVA, Pre-IOP and 1m-BCVA, and Pre-IOP and Change in BCVA

**Figure 2:**
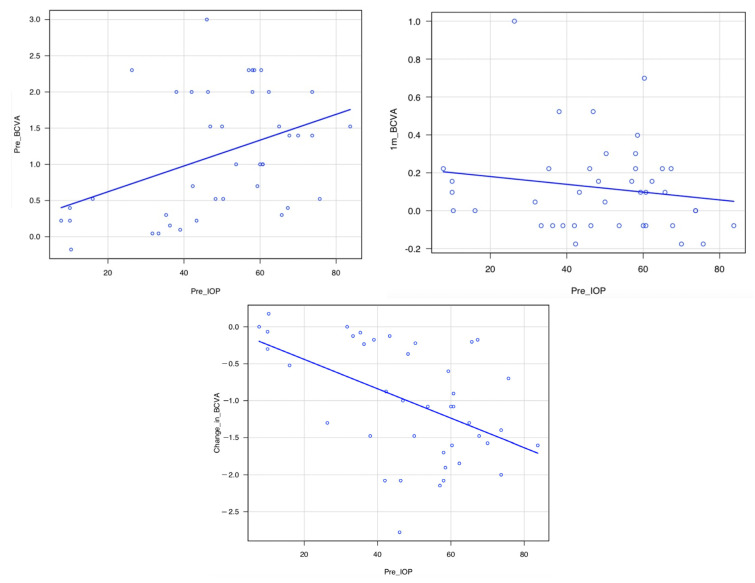
Correlation between Pre-IOP and Pre-BCVA, Pre-IOP and 1m-BCVA, and Pre-IOP and Change in BCVA. The pre-IOP and pre-BCVA and pre-IOP and change in BCVA for all 41 eyes were significantly correlated (*p* = 0.0080, r = 0.41, *p* = 0.0010, r = −0.50, respectively); however, pre-IOP and 1m-BCVA were not significantly correlated (*p* = 0.31). Abbreviations: BCVA: LogMAR-converted best corrected visual acuity, IOP: intraocular pressure (mmHg), Pre-BCVA: BCVA at the first visit, Pre-IOP: IOP at the first visit (mmHg), 1m-BCVA: BCVA at 1 month, and Change in BCVA: Change in BCVA from first visit to 1 month.

The pre-IOP and pre-BCVA and pre-IOP and change in BCVA for all 41 eyes were significantly correlated (*p* = 0.0080, r = 0.41, *p* = 0.0010, r = −0.50, respectively); however, pre-IOP and 1m-BCVA were not significantly correlated (*p* = 0.31) (Table 4) (Figure 2).

## 4. Discussion

Although the follow-up period was only one month in this study, early phacoemulsification as a primary treatment for APAC resulted in rapid visual improvement, retention of the number of corneal endothelial cells, and good IOP control without glaucoma treatment in all 41 eyes.

Few studies have reported on the postoperative outcomes after phacoemulsification without LPI as the primary treatment for APAC in more than 40 cases [3,7]. All patients in these reports received preoperative IOP-lowering medication or underwent preoperative postural restriction to remove the pupillary block.

The IOP at the beginning of the surgery in the present cases was on average higher than that in previous reports [3,4,5], where the IOP at the beginning of the surgery was less than 35, 21, and 30 mmHg due to the use of preoperative IOP-lowering medications. However, in the current study, early phacoemulsification with or without the use of IOP-lowering medications resulted in a good visual prognosis, similar to that in previous reports [3,4,5]. No serious complications were observed, and reoperation was not required. However, preoperative IOP-lowering medications can improve corneal edema, increase intraoperative visibility, and reduce surgical difficulty. We performed surgery without preoperative medications in some cases due the following reasons: Firstly, we were hesitant to use systemic administration of carbonic anhydrase inhibitors and some glaucoma eye drops such as β blockers in patients with poor systemic conditions such as renal, cardiac, and respiratory dysfunction. Secondly, there are patients whose APAC does not resolve with medication alone or whose IOP does not decrease; in such cases, optic neuropathy due to ultrahigh IOP worsens on an hourly basis. Thirdly, corneal endothelial damage can be a problem in LPI [8,9]. Finally, we were able to operate on patients with APAC immediately after their visit to our hospital. In fact, average time from onset to operation was 3.3 h in the Medication (−) group, with a track record of performing safe surgery without severe complication such as suprachoroidal hemorrhage.

Previous reports have compared the results of phacoemulsification and LPI for APAC [3,4,5,6], and a review [10] has summarized these results. Despite the limitations of surgical difficulty, phacoemulsification was reported to be a better treatment option than LPI with respect to long-term IOP control and cost; however, the appropriate timing of surgery remains unclear [10] (Table 5).

There have been many reports on the problems associated with LPI. LPI can cause corneal endothelial damage [9,10]. Bullous keratopathy secondary (BK) to LPI is the second most common cause of BK, accounting for 23.4% of BK cases that required total keratoplasties between 1999 and 2001 in Japan [12]. The extent of PAS increased after LPI for APAC [11]. However, LPI for APAC tends to cause a long-term re-elevation of IOP, often requiring medications or reoperation to control IOP [13,14,15]. LPI for APAC can also cause long-term glaucomatous optic neuropathy [16], and unoperated cataracts cause a decrease in visual acuity [16] (Table 6).

In contrast, in the current study, we believe that early phacoemulsification was able to unblock lens-induced pupillary block, achieve angle opening and anterior chamber depth [18], prevent Schlemm’s canal endothelial damage due to persistent contact between the trabecular meshwork and iris, and prevent subsequent Schlemm’s canal obstruction and damage [19]. These are thought to provide good IOP control. Although advanced surgical techniques are required, we believe that phacoemulsification is becoming the primary treatment for APAC considering recent improvements in the safety of cataract surgery [20].

In the current study, IOP-lowering medications or reoperation due to re-elevation of IOP were not required one month after surgery. Husain et al. [4] required trabeculectomy the day after phacoemulsification in 1 out of 19 cases of PEA-IOL; however, the other phacoemulsification cases did not require reoperation for 2 years. Lam et al. [5] also reported good results with no requirement of reoperation at 1.5 years in the phacoemulsification group. Considering the results of these RCTs on phacoemulsification, it can be assumed that if IOP does not increase after phacoemulsification in the short term, good IOP control can be achieved in the long term for at least 1.5–2 years.

The time from APAC onset to phacoemulsification in the previous studies was approximately 0.1 to 10 days [3,4,5,6]. Reoperation was required in the phacoemulsification group because of poor IOP control in the reports with a time from APAC onset to phacoemulsification of approximately 2 to 10 days [3,4]. In contrast, reoperation was not required in the reports with a time from APAC onset to phacoemulsification of approximately 0.1 to 10 days [5,6].

In the present study, the average time from APAC onset to surgery was approximately 2 days, and reoperation was not required until at least 1 month after surgery. Although the appropriate time for phacoemulsification for APAC is still unclear [8], considering the previous reports [3,4,5,6] and the results of our study, phacoemulsification performed within 1 to 2 days can prevent irreversible Schlemm’s canal endothelial damage, Schlemm’s canal obstruction, and trabecular meshwork damage, leading to good long-term IOP control.

The higher the preoperative IOP, the poorer the preoperative BCVA. However, the higher the preoperative IOP, the greater the improvement in BCVA, possibly because the low preoperative visual acuity was not due to irreversible central visual field defects caused by glaucomatous optic neuropathy but due to temporary corneal edema caused by high IOP and cataract.

We believe that rapid phacoemulsification contributes to the prevention of central visual field damage by promptly lowering the IOP.

Pupillary block is the most frequent and important mechanism responsible for angle closure [21], though, it is not the only mechanism involved. For example, in the plateau iris configuration, the iris is held anteriorly by the ciliary processes, but a pupillary block component may also be present [22]. The subjects included in this study had an average age of about 73 years. However, plateau iris is a common cause of angle closure in younger patients, predominantly in women 30–50 years of age [23]. A study reported that clear lens extraction was effective in treating plateau iris in a young patient, where LPI could not be performed [17]. Additionally, LPI can cause corneal endothelial damage [9,10]; hence, we believe that primary phacoemulsification should also be an option for APAC in young patients.

This study has several limitations. Phacoemulsification for APAC is a difficult procedure and requires a certain level of skill from the surgeon as it involves a shallow anterior chamber [24], weakness of the zonular fibers [25], or corneal edema [26]. Moreover, the long-term prognosis including necessity of anti-glaucoma medication beyond one month is unknown, and comparisons with other treatment modalities, such as LPI, were not performed. Furthermore, due to perioperative corneal edema or the patients’ symptoms, such as headache or nausea, precise evaluation of the preoperative and postoperative PAS range and the effect of GSL was not possible. Lastly, the visual field and optic nerve fiber layer damage were not evaluated before and after surgery.

Further studies are needed to examine the long-term surgical outcomes in a larger number of cases.

## 5. Conclusions

Rapid primary phacoemulsification for APAC with or without preoperative IOP-lowering treatment resulted in good visual improvement, retention of corneal endothelial cells, and good IOP control without the need for reoperation.

## Figures and Tables

**Table 1:** Summary of results for the current study.

Parameters	Mean ± SD	Range	*p*-Value
Number of eyes	41	-	
Male	9	-	
Female	32	-	0.0004 ^†^
Age (years)	73.7 ± 8.7	49–93	
Time from onset to operation (h)	48.0 ± 46.6	5–175	
Time from arrival to operation (h)	5.9 ± 7.3	1–31	
Operation time (m)	20.2 ± 7.9	11.8–44.8	
BCVA (LogMAR)			
First visit	1.13 ± 0.85	−0.18 to 3	
1 day	0.56 ± 0.72 *	−0.18 to 2.30	0.0002
1 week	0.27 ± 0.42 *	−0.18 to 2	<0.0001
1 month	0.12 ± 0.24 *	−0.18 to 1	<0.0001
IOP (mmhg)			
First visit	48.8 ± 19.4	7.7–83.7	
1 day	14.6 ± 8.1 *	6.3–37.3	<0.0001
1 week	12.8 ± 3.0 *	8–19.7	<0.0001
1 month	12.5 ± 2.6 *	9–21.3	<0.0001
CECs (/mm^2^)			
First visit	2266 ± 796	571–3425	
1 month	2419 ± 704	874–3497	0.301
Medication)			
First visit ((+) 26, (−) 15 eyes)	1.4 ± 1.6	0–6	
1 month	0 ± 0 *	0–0	<0.0001

* Significantly different from the first visit (*p* < 0.05). ^†^ Sex differences in all cases. Abbreviations: h, hour; m, minute; BCVA (LogMAR), LogMAR-converted best corrected visual acuity; IOP, intraocular pressure; CECs, number of corneal epithelial cells, and medication, number of glaucoma medication.

**Table 2:** Summary of subgroup analysis.

Parameters	Medication (+)	Medication (−)	*p*-Value
Number of eyes	26	15	
Male	6	3	
Female	20	12	1 ^†^
Age (years)	73.0 ± 8.5	74.9 ± 8.9	0.69
Time from onset to operation (h)	48.5 ± 51.9	47.3 ± 53.8	0.19
Time from arrival to operation (h)	7.3 ± 8.5	3.3 ± 3.5	0.06
Operation time (m)	20.0 ± 7.9	20.3 ± 7.9	0.87
BCVA (LogMAR)			
First visit	1.29 ± 0.81	0.86 ± 0.80	0.12
1 day	0.58 ± 0.70	0.53 ± 0.72	0.94
1 week	0.34 ± 0.47	0.16 ± 0.23	0.30
1 month	0.17 ± 0.27	0.03 ± 0.14	0.15
IOP (mmhg)			
First visit	48.3 ± 20.4	49.7 ± 16.7	1.00
1 day	13.6 ± 6.5	16.2 ± 9.9	0.27
1 week	12.6 ± 3.0	13.2 ± 3.0	0.60
1 month	12.3 ± 2.5	12.8 ± 2.8	0.45
Cecs (/mm^2^)			
First visit	2138 ± 918	2465 ± 495	0.41
1 month	2432 ± 772	2400 ± 585	0.69
Medication)			
First visit *	2.2 ± 1.4	0 ± 0	<0.0001
1 month	0 ± 0	0 ± 0	Not a number

* Significant difference between the two groups (*p* < 0.05). ^†^ Difference in sex ratio between the two groups. Medication (+): Group of patients received intraocular pressure-lowering medication before surgery. Medication (−): Group of patients did not receive intraocular pressure-lowering medication before surgery. Abbreviations: h, hour; m, minute; BCVA (LogMAR), LogMAR-converted best corrected visual acuity; IOP, intraocular pressure; CECs, number of corneal epithelial cells, and medication, number of glaucoma medication.

**Table 3:** Summary of surgical complications.

Parameters	Medication (+)	Medication (−)
Preoperative corneal edema	20	10
Intraoperative iris prolapse	3	0
Intraoperative hyphema	3	0
Zinn’s zonule rupture within 45 degrees.	0	1
Crack in CCC	1	0
Iris dissection at 45 degrees	1	0
Postoperative fibrin in anterior chamber	2	9
Postoperative hyphema	1	3
Reoperation	0	0

Medication (+): Twenty-six patients received intraocular pressure-lowering medication before surgery. Medication (−): Fifteen patients did not receive intraocular pressure-lowering medication before surgery. Abbreviations: CCC, continuous curvilinear capsulorrhexis.

**Table 4:** Correlation between Pre-IOP and Pre-BCVA, Pre-IOP and 1m-BCVA, and Pre-IOP and Change in BCVA.

	Pre-BCVA	Change in BCVA	1m-BCVA
Pre-IOP	* *p* = 0.0080, r = 0.41	* *p* = 0.0010, r = −0.50	*p* = 0.31, r = −0.16

Abbreviations: BCVA, LogMAR-converted best corrected visual acuity; IOP, intra ocular pressure; Pre-BCVA, BCVA at the first visit; Pre-IOP, IOP at the first visit; 1m-BCVA, BCVA at 1 month; and Change in BCVA, Change of BCVA from first visit to 1 month; *, Significant correlation (*p* < 0.05).

**Table 5:** Comparison between previous reports about PEA-IOL and our study.

Authors	Study Design	Preoperative IOP Lowering Medication	Average Follow-Up (Months)	No. of Patients	Type of Treatments	Time from Onset to Operation	Preoperative IOP (mmHg)	Postoperative IOP (mmHg)	No. of Additional Surgery Required (%)
Lam et al. [5]	RCT	+	18	31	Phaco/IOL	Onset to consultation 3.1 ± 3.7 (days). Consultation to abortion of attack 11.4 ± 9.0 (h). Abortion of attack to PEAIOL 5.7 ± 3.3 (days)	59.7 ± 8.71	12.6 ± 1.9	0
				31	LPI	Onset to consultation 2.2 ± 2.5 (days). Consultation to abortion of attack 9.1 ± 6.9 (h). Abortion of attack to LPI 4.3 ± 2.7 (days)	57.9 ± 11.8	15.0 ± 3.4	4 (12.9) repeated LPI
Husain et al. [4]	RCT	+	24	19	Phaco/IOL	Onset to consultation 2.8 (days). Consultation to PEA-IOL 5–7 (days)	57.4 16.9	15.4 7.7	1 (5.3) TBx on day 1
				18	LPI	Onset to consultation 7.4 (days). Consultation to LPI 3 (days)	55.8 13.2	13.7 6.1	6 (33.3) phaco TBx, 1 (5.6) repeated LPI
Jacobi et al. [3]	Non-RCT	+	10.2 ± 3.4	43	Phaco/IOL	Onset to PEA-IOL 2.1 ± 0.9 (days)	40.5 ± 7.6	17.80 ± 3.40	2 (4.6) filtration procedure, 3 (6.9) CPC
				32	SPI	Onset to SPI 2.0 ± 0.8 (days)	39.7 ± 7.8	20.10 ± 4.20	11 (34.3) Phaco/IOL, 5 (15.6) filtration procedure, 4 (12.5) CPC
Imaizumi et al. [6]	NM	+	6	18	Phaco/IOL	Onset to PEA-IOL 0.11 ± 0.32 (days)	48.9 ± 13.9	13.0 ± 3.1	0
				8	LPI→Phaco/IOL	Onset to PEA-IOL 90 ± 90(months)	17.0 ± 3.9	13.5 ± 1.1	0
Moghimi et al. [11]	Prospective non-randomised	+	18.5 ± 5.2	20	LPI→Phaco/IOL	Onset to consultation 4.6 ±4.7(days). Abortion of attack and LPI 2.1 ± 1.6 (days). Abortion of attack and PEA-IOL 23.6 ± 9.2(days)	54.0 ± 9.4	13.90 ± 2.17	0
				15	LPI only	Onset to consultation 5.1 ± 4.8(days). Abortion of attack and LPI 1.7 ± 1.5(days).	57.1 ± 10.2	17.80 ± 4.16	3 (20.0) underwent phaco/IOL and 2 (13.3) underwent phaco TBx.
Our study	Retrospective, case series	+/−	1	41	Phaco/IOL(-GSL)	Onset to PEA-IOL 2.0 ± 1.9 (days)	48.8 ± 19.4	12.5 ± 2.6	0

Abbreviations: RCT, randomized control trial; NM, not mentioned; phaco/IOL, phacoemulsification with intraocular lens insertion; LPI, laser peripheral iridotomy; GSL, goniosynechialysis; TBx, trabeculectomy; phaco TBx, combined phacotrabeculectomy; CPC, cyclophotocoagulation; LPI, phaco/IOL, laser peripheral iridotomy followed by phacoemulsification with intraocular lens insertion.

**Table 6:** Comparison between previous reports about LPI and our study.

Authors	Study Design	Average Follow-Up (Months)	No. of Eyes (Patients)	Type of Treatments	Age	Time from Onset to Operation	Preoperative IOP (mmHg)	Postoperative IOP (mmHg)	No. of Additional Surgery Required (%)
Aung et al. [13]	Retrospective	50.3 (9–107)	110 (96)	LPI	63.7 (39–92)	Onset to consultation 6.5 (days). Consutation to LPI < 3 (days)	52.8 (28–80)	NM	36 (32.7) TBx
Aung et al. [16]	Cross-sectional	75.6 ± 18.0 (49.2–121.2)	90 (90)	LPI	62.0 ± 9.0 (43–89)	Onset to consultation 31 eyes < 1 day, 20 eyes 1–3 days, 36 eyes > 3 days	NM	15.4	34 (37.8) filtering surgery
Lim et al. [14]	Prospective observational case series	12	44(44)	LPI	60.2 ± 10.7 (35–99)	Onset to consultation 35.8 ± 56.4(h). Consultation to LPI 2.5 ± 1.3(days)	53.3 ± 15.2 (26–78)	NM	NM
Tan et al. [17]	Retrospective	27.3 ± 16.2	42 (41)	LPI	59.6 ± 11.3 (37–85)	Onset to consultation 28.2 (h), consultation to LPI < 24 (h)	55.0 ± 12	13.3 ± 2.92	12 (28.6) Phaco/IOL, 7 (16.7) Phaco TBx, 1 (2.4) Phaco GDD
Lai et al. [10]	RCT	16.4 ± 5.6 (7–29)	38 (32)	Medications→LPI	66.5 ± 8.5	Onset to treatment 29.7 ± 23.8 (h). Consultation to LPI < 48 (h)	57.1 ± 9.2 (40–74)	14.7 ± 4.6 (8–32)	1 (2.6) Phaco/IOL
		15.0 ± 6.0 (6–36)	41 (39)	ALPI→LPI	70.0 ± 10.5	Onset to treatment 41.6 ± 47.6 (h). Consultation to LPI < 48 (h)	61.2 ± 10.6 (40–79)	13.6 ± 2.7 (9–21)	1 (2.4) Phaco/IOL for CACG, 2 (4.9) Phaco/IOL for cataract
Our study	Retrospective, case series	1	41(41)	Phaco/IOL(-GSL)	73.7 ± 8.7 (49–93)	Onset to PEA-IOL 2.0 ± 1.9 (days)	48.8 ± 19.4	12.5 ± 2.6	0

Abbreviations: RCT, randomized control trial; NM, not mentioned; phaco/IOL, phacoemulsification with intraocular lens insertion; LPI, laser peripheral iridotomy; ALPI, argon laser peripheral iridoplasty; GSL, goniosynechialysis; TBx, trabeculectomy; LPI→phaco/IOL, laser peripheral iridotomy followed by phacoemulsification with intraocular lens insertion; phacoGOD, combined aqueous shunt device and phacoemulsification; and CACG, chronic angle closure glaucoma.

## Data Availability

The data presented in this study are available on request from the corresponding author.

## References

[1] Tham Y.C., Li X., Wong T.Y., Quigley H.A., Aung T., Cheng C.Y. (2014). Global prevalence of glaucoma and projections of glaucoma burden through 2040: A systematic review and meta-analysis. Ophthalmology.

[2] Burton M.J., Ramke J., Marques A.P., Bourne R.R.A., Congdon N., Jones I., Ah Tong B.A.M., Arunga S., Bachani D., Bascaran C. (2021). The Lancet Global Health Commission on Global Eye Health: Vision beyond 2020. Lancet Glob. Health.

[3] Jacobi P.C., Dietlein T.S., Lüke C., Engels B., Krieglstein G.K. (2002). Primary phacoemulsification and intraocular lens implantation for acute angle-closure glaucoma. Ophthalmology.

[4] Husain R., Gazzard G., Aung T., Chen Y., Padmanabhan V., Oen F.T., Seah S.K., Hoh S.T. (2012). Initial management of acute primary angle closure: A randomized trial comparing phacoemulsification with laser peripheral iridotomy. Ophthalmology.

[5] Lam D.S., Leung D.Y., Tham C.C., Li F.C., Kwong Y.Y., Chiu T.Y., Fan D.S. (2008). Randomized trial of early phacoemulsification versus peripheral iridotomy to prevent intraocular pressure rise after acute primary angle closure. Ophthalmology.

[6] Imaizumi M., Takaki Y., Yamashita H. (2006). Phacoemulsification and intraocular lens implantation for acute angle closure not treated or previously treated by laser iridotomy. J. Cataract Refract. Surg..

[7] Azuara-Blanco A., Burr J., Ramsay C., Cooper D., Foster P.J., Friedman D.S., Scotland G., Javanbakht M., Cochrane C., Norrie J. (2016). Effectiveness of early lens extraction for the treatment of primary angle-closure glaucoma (EAGLE): A randomised controlled trial. Lancet.

[8] Lam D.S., Lai J.S., Tham C.C., Chua J.K., Poon A.S. (2002). Argon laser peripheral iridoplasty versus conventional systemic medical therapy in treatment of acute primary angle-closure glaucoma: A prospective, randomized, controlled trial. Ophthalmology.

[9] Lai J.S., Tham C.C., Chua J.K., Poon A.S., Chan J.C., Lam S.W., Lam D.S. (2006). To compare argon laser peripheral iridoplasty (ALPI) against systemic medications in treatment of acute primary angle-closure: Mid-term results. Eye.

[10] Chan P.P., Pang J.C., Tham C.C. (2019). Acute primary angle closure-treatment strategies, evidences and economical considerations. Eye.

[11] Moghimi S., Hashemian H., Chen R., Johari M., Mohammadi M., Lin S.C. (2015). Early phacoemulsification in patients with acute primary angle closure. J. Curr. Ophthalmol..

[12] Shimazaki J., Amano S., Uno T., Maeda N., Yokoi N., Japan Bullous Keratopathy Study Group (2007). National survey on bullous keratopathy in Japan. Cornea.

[13] Aung T., Ang L.P., Chan S.P., Chew P.T. (2001). Acute primary angle-closure: Long-term intraocular pressure outcome in Asian eyes. Am. J. Ophthalmol..

[14] Lim L.S., Aung T., Husain R., Wu Y.J., Gazzard G., Seah S.K. (2004). Acute primary angle closure: Configuration of the drainage angle in the first year after laser peripheral iridotomy. Ophthalmology.

[15] Choong Y.F., Irfan S., Menage M.J. (1999). Acute angle closure glaucoma: An evaluation of a protocol for acute treatment. Eye.

[16] Aung T., Friedman D.S., Chew P.T., Ang L.P., Gazzard G., Lai Y.F., Yip L., Lai H., Quigley H., Seah S.K. (2004). Long-term outcomes in Asians after acute primary angle closure. Ophthalmology.

[17] Rao A. (2013). Clear lens extraction in plateau iris with bilateral acute angle closure in young. J. Glaucoma..

[18] Li S.W., Chen Y., Wu Q., Lu B., Wang W.Q., Fang J. (2015). Angle parameter changes of phacoemulsification and combined phacotrabeculectomy for acute primary angle closure. Int. J. Ophthalmol..

[19] Hamanaka T., Kasahara K., Takemura T. (2011). Histopathology of the trabecular meshwork and Schlemm’s canal in primary angle-closure glaucoma. Investig. Ophthalmol. Vis. Sci..

[20] Olson R.J. (2018). Cataract surgery from 1918 to the present and future-just imagine!. Am. J. Ophthalmol..

[21] Sun X., Dai Y., Chen Y., Yu D.Y., Cringle S.J., Chen J., Kong X., Wang X., Jiang C. (2017). Primary angle closure glaucoma: What we know and what we don’t know. Prog. Retin. Eye Res..

[22] Wand M., Grant W.M., Simmons R.J., Hutchinson B.T. (1977). Plateau iris syndrome. Trans. Sect. Ophthalmol. Am. Acad. Ophthalmol. Otolaryngol..

[23] Ritch R., Chang B.M., Liebmann J.M. (2003). Angle closure in younger patients. Ophthalmology.

[24] Hsia Y., Su C.C., Wang T.H., Huang J.Y. (2021). Posture-related changes of intraocular pressure in patients with acute primary angle closure. Investig. Ophthalmol. Vis. Sci..

[25] Kono M., Ishida A., Ichioka S., Matsuo M., Shimizu H., Tanito M. (2021). Aphakic pupillary block by an intact anterior vitreous membrane after total lens extraction by phacoemulsification. Case Rep. Ophthalmol..

[26] Varadaraj V., Sengupta S., Palaniswamy K., Srinivasan K., Kader M.A., Raman G., Reddy S., Ramulu P.Y., Venkatesh R. (2017). Evaluation of angle closure as a risk factor for reduced corneal endothelial cell density. J. Glaucoma..

